# Prevalence of suboccipital and intradural vertebral artery variants: a systematic review with meta-analysis

**DOI:** 10.1007/s00234-025-03674-2

**Published:** 2025-06-16

**Authors:** George Triantafyllou, Panagiotis Papadopoulos-Manoralarakis, Rǎzvan Costin Tudose, Mugurel Constantin Rusu, George Tsakotos, Maria Piagkou

**Affiliations:** 1 Department of Anatomy, School of Medicine, Faculty of Health Sciences, National and Kapodistrian University of Athens, Athens, Greece; 2 Department of Neurosurgery, General Hospital of Nikaia-Piraeus, Athens, Greece; 3 Division of Anatomy, Department 1, Faculty of Dentistry, Carol Davila University of Medicine and Pharmacy, Bucharest, Romania

**Keywords:** Vertebral artery, Variation, Suboccipital segment, Intradural segment, Neuroradiology, Evidence-based anatomy, Meta-analysis

## Abstract

**Purpose:**

The current systematic review, along with a meta-analysis, aims to present and calculate the pooled prevalence of the morphological variants of the vertebral artery (VA) within the suboccipital (V3) and intradural (V4) segments.

**Methods:**

According to the most recent guidelines, the systematic review was conducted using four online databases. Eligible studies were extracted, and a meta-analysis was performed utilizing R programming software. A subgroup analysis was conducted based on nationality and imaging techniques.

**Results:**

The systematic review revealed thirty-two (32) studies involving 176,391 patients. The pooled prevalence of VA fenestration was established at 0.30% (95% CI: 0.14–0.51). Furthermore, the persistent first intersegmental artery (PFIA) was estimated to have a pooled prevalence of 1.17% (95% CI: 0.36–2.34), wherein the imaging technique and nationality served as a significant moderator. An aberrant origin of the posterior inferior cerebellar artery from the V3 segment was identified, with a pooled prevalence of 2.69% (95% CI: 1.13–4.82). Additionally, the pooled VA dominance, hypoplasia, and atresia prevalences were recorded at 27.45%, 13.41%, and 5.39%, respectively.

**Conclusions:**

Magnetic resonance or computed tomography angiographies can accurately illustrate the distal VA’s diverse anatomical configurations. Specific variants hold substantial clinical significance, as they are correlated with posterior circulation cerebrovascular incidents and may complicate posterior approaches to the craniocervical region.

## Introduction

The morphological variability of the cerebral arterial circle has been the subject of extensive investigation, primarily through imaging studies that appropriately document cerebral morphological variants and their significance for clinicians, including neurosurgeons and neuroradiologists [1–3].

The cerebral arterial circle is constituted by anastomoses among the branches of the internal carotid and vertebrobasilar arterial systems. The vertebral artery (VA) originates bilaterally from the first part of the subclavian artery (first segment - V1). Subsequently, both VAs ascend through the transverse foramen (TF) of the sixth cervical vertebra (C6) until reaching the TF of the second cervical vertebra (C2) (second segment - V2). The suboccipital (third segment - V3) segment pertains to the pathway from C2 to the dura matter at the foramen magnum (FM), and the last segment, the intradural one (fourth segment - V4), refers to the course extending from the FM to the confluence with the contralateral VA, forming the basilar artery (BA). The V4 segment gives rise to several branches, including the posterior inferior cerebellar artery (PICA) [4, 5].

Numerous morphological variants have been documented for each VA segment. Studies utilizing computed tomography and magnetic resonance angiography (CTA and MRA) either concentrate on the VA origin (V1) and entry point into the TF (V2) [6] or examine the suboccipital (V3) and intradural (V4) segments [7]. Furthermore, limited variations are also reported, including persistent embryological anastomoses between the internal carotid and vertebrobasilar arterial systems [8].

Each segment of the VA has few significant clinical implications, especially for neuroradiologists and neurosurgeons. Variants of the origin and proximal course of the VA can be dangerous during surgical procedures of the aortic arch or neck region, as well as during angiographies [6]. The suboccipital segment is implicated during approaches at the atlanto-occipital junction and the intradural segment during skull base surgeries. Variants of these segments can significantly complicate the surgical procedures. Nevertheless, variations can increase the risk of certain pathologies, such as the atresia and hypoplasia that are linked with hemodynamic insufficiency. Thus, adequate knowledge of the vascular anatomy of the VA with the use of imaging techniques is of outmost importance [9–12].

Several comprehensive reviews have investigated the VA variations [9–12]. A prior evidence-based meta-analysis documented the morphological variability of V1 and V2 segments [13]. As a result, the objective of the current evidence-based meta-analysis is to systematically examine the V3 (suboccipital) and V4 (intradural) segments to complete the systematic study of the VA segments and facilitate further systematic original research encompassing the entirety of the VA (all segments).

## Materials and methods

The systematic review with meta-analysis followed the guidelines established by the Evidence-based Anatomy Workgroup for anatomical meta-analysis [14] and the PRISMA 2020 for systematic reviews [15]. This study’s protocol was not registered in any online database. The figures were obtained from an archive of the General Hospital of Nikaia-Piraeus after ethical approval (number of approval: 56485; date: 13/11/2024).

A literature search was conducted using the online databases PubMed, Google Scholar, Scopus, and Web of Science until December 2024. The following terms were employed in various combinations: “vertebral artery,” “intracranial vertebral artery,” “variation,” “vertebral artery fenestration,” “persistent first intersegmental artery,” “C2 segmental type vertebral artery,” “posterior inferior cerebellar artery,” “imaging study,” and “radiological study.” Additionally, the references of all included articles were assessed, grey literature was explored, and a thorough search of key anatomical journals (*Annals of Anatomy*,* Clinical Anatomy*,* Journal of Anatomy*,* Anatomical Record*,* Surgical and Radiological Anatomy*,* Folia Morphologica*,* European Journal of Anatomy*,* Morphologie*,* Anatomical Science International*,* Anatomy and Cell Biology*) was conducted. The inclusion criteria comprised studies that reported the prevalence of intracranial VA variations via CTA or MRA. Case reports, conference abstracts, animal studies, and studies presenting irrelevant or insufficient data were excluded. The examination of the VA in its suboccipital (V3) and intracranial (V4) segments recorded the subsequent morphological variants based on their aggregated prevalence, arranged in order of decreasing frequency prevalence. In the V3 segment, the persistent first intersegmental artery (PFIA) or C2 segmental-type VA was identified, which represents an embryological artery associated with the carotid-basilar anastomoses and the aberrant origin of the PICA in an extradural context. In the V4 segment, the findings included VA fenestration, hypoplasia (defined as a VA diameter of less than 1 mm), dominance (characterized by a difference in VA diameter exceeding 2 mm, as established by Isaji et al. [16]), and atresia (where the vessel continues as PICA).

Two independent reviewers (GTr and PPM) searched the literature and extracted the data into Microsoft Excel sheets. The results were compared, and the other authors resolved any discrepancies. The Anatomical Quality Assurance (AQUA) tool, developed by the Evidence-based Anatomy Workgroup for anatomical reviews [17], was used to assess each article’s risk of bias.

A statistical meta-analysis was performed using the open-source R programming language and RStudio software (version 4.3.2), utilizing the “meta” and “metafor” packages by a single investigator (GTr). The pooled prevalence was calculated using inverse variance and random effects models. The proportions (prevalence) meta-analysis was conducted using the Freeman-Tukey double arcsine transformation, the DerSimonian-Laird estimator for the between-study variance tau², and the Jackson method for the confidence interval of tau² and tau. Furthermore, several subgroup analyses were conducted to identify variables (geographic distribution, laterality, or imaging technique) affecting the estimated pooled prevalence and mean. A p-value of less than 0.05 was deemed statistically significant. Cochran’s Q statistic was utilized to assess the presence of heterogeneity across studies, while the Higgins I² statistic quantified this heterogeneity. Cochran’s Q p-value < 0.10 was considered significant. Higgins I² values between 0 and 40% were regarded as negligible, 30–60% as moderate heterogeneity, 50–90% as substantial heterogeneity, and 75–100% as considerable heterogeneity. To investigate the small-study effect (the phenomenon where smaller studies may exhibit different effects than larger ones), the DOI plot with the LFK index was generated for proportional parameters [18].

## Results

### Search analysis

**Fig. 1:**
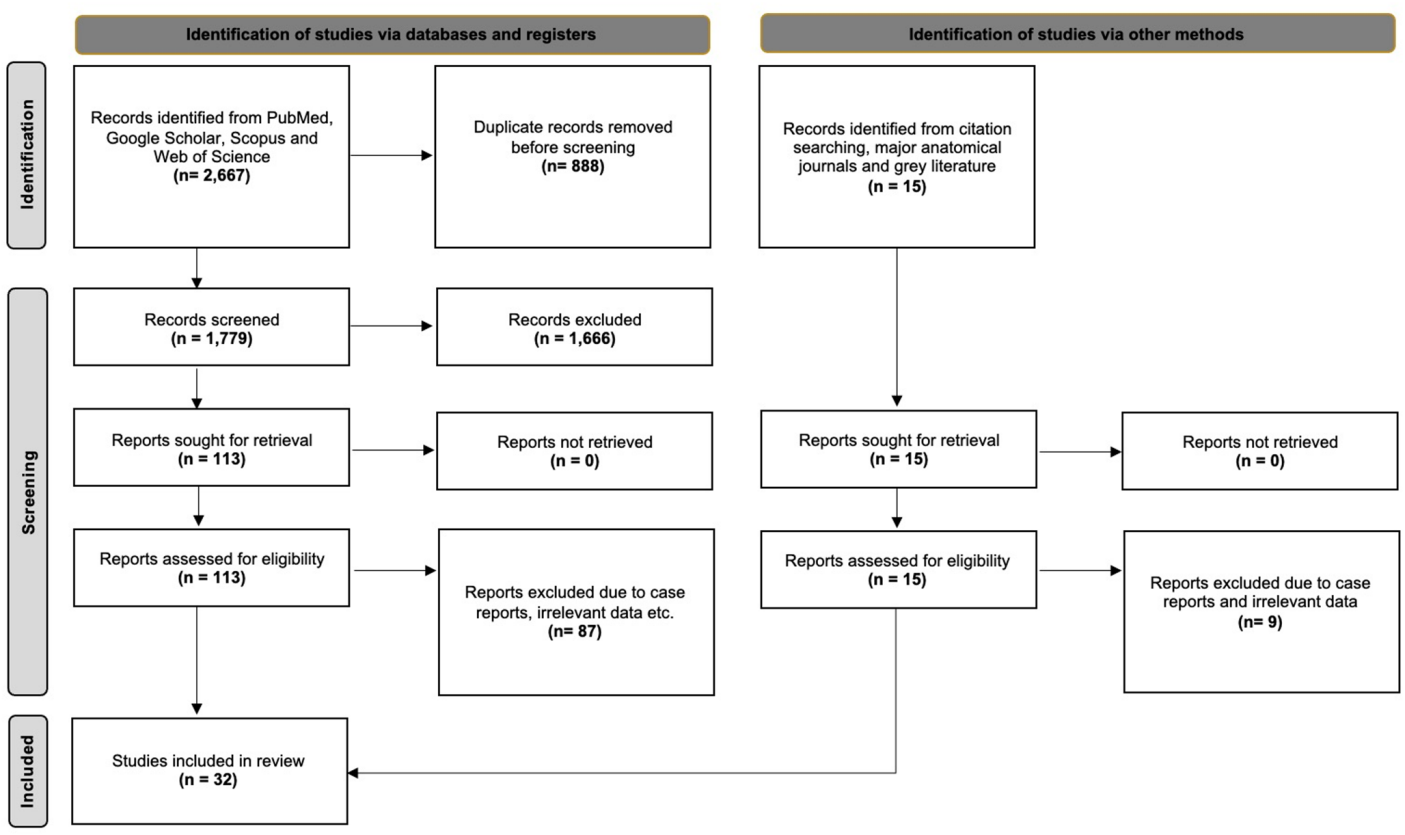
The search analysis flow chart according to the PRISMA 2020 guidelines

The database search yielded 2,667 articles exported to Mendeley version 2.10.9 (Elsevier, London). After excluding duplicate and irrelevant papers through title and abstract screening, 113 studies were subjected to full-text retrieval and screening. Ultimately, 26 studies were deemed eligible for systematic review. Additionally, six studies were identified through our secondary investigation, which encompassed references, grey literature, and a hands-on search of anatomical journals. Hence, 32 studies were included in our systematic review with meta-analysis. Figure 1 summarizes the flow diagram of our search analysis by the PRISMA 2020 guidelines.

### Eligible characteristics of eligible studies

Thirty-two (32) studies were included, comprising 176,391 patients. The mean sample size per article was 5,512.22 patients. Concerning the imaging technique used, twenty-five (25) analyses were conducted using CTA scans, six (6) studies were based on MRA scans, and one utilized angiograms. Concerning the included population, twenty-one (21) studies were associated with the Asian population, seven (7) with the European population, three (3) with the American, and one (1) with the African population. The characteristics of the included studies are summarized in Table 1.

**Table 1:** The characteristics of the eligible studies, including their risk of bias assessment based on the AQUA tool [17]

Study	Year	Population	Type of Study	Patients	Risk of Bias
Tokuda et al. [19]	1985	Asia	Angiograms	300	High
Parmar et al. [20]	2005	Asia	MRA (3 Tesla)	843	Low
Hong et al. [21]	2008	Asia	CTA (slice thickness: 1.0 mm)	1013	Low
Yamaguchi et al. [22]	2008	Asia	CTA (16-slices; slice thickness: 0.625 mm)	140	Low
Duan et al. [23]	2010	Asia	CTA (slice thickness: 1.25 mm)	98	Low
Uchino et al. [24]	2012a	Asia	MRA (1.5 Tesla)	3327	Low
Uchino et al. [7]	2012b	Asia	MRA (1.5 Tesla)	2739	Low
Kovac et al. [25]	2014	Europe	CTA (16-slices; slice thickness: 0.625 mm)	512	Low
O’Donnell et al. [26]	2014	America	CTA (slice thickness: 1.0 mm)	975	Low
Pekcevik et al. [27]	2014	Asia	CTA (64 slices; slice thickness: 0.5 mm)	341	Low
Wakao et al. [28]	2014	America	CTA (64-slices)	480	Low
Van Rooij et al. [29]	2015	Europe	CTA	140	High
Zampakis et al. [30]	2015	Europe	CTA (16-slices; slice thickness: 1.25 mm)	1739	Low
Fortuniak et al. [31]	2016	Europe	CTA (64-slices; slice thickness: 0.625 mm)	1800	Low
Hong et al. [32]	2016	Asia	CTA	147	High
Kim [33]	2016	Asia	CTA (64 slices, slice thickness: 0.5 mm)	3067	Low
Dzierzanowski et al. [34]	2017	Europe	CTA (64-slices; slice thickness: 0.625 mm)	104	Low
Liu et al. [35]	2017	Asia	MRA	160	High
Vanek et al. [36]	2017	Europe	CTA (slice thickness: 0.8 mm)	511	Low
Isaji et al. [16]	2018	Asia	CTA (64-slices)	153	Low
Xu et al. [37]	2018	Asia	CTA (slice thickness: 1.0 mm)	139	High
Zhang et al. [38]	2018	Asia	CTA (64-slices)	200	High
Kim [39]	2018	Asia	CTA (128 slices; slice thickness: 0.5 mm)	3386	Low
Arslan et al. [39]	2019	Asia	CTA (64 slices; slice thickness: 0.5 mm)	200	High
Li et al. [40]	2019	Asia	CTA (64-slice)	120	High
D’Sa et al. [41]	2020	America	CTA and MRA	44,759	High
Omotoso et al. [42]	2021	Africa	CTA (64-slices; slice thickness: 0.625 mm)	554	Low
Wang et al. [43]	2021	Asia	CTA (64 slices; slice thickness: 0.9 mm)	589	Low
Dharshini et al. [44]	2022	Asia	CTA	50	High
Zhu et al. [45]	2022	Asia	MRA	2528	High
Davidoiu et al. [46]	2024	Europe	CTA (32 slices; slice thickness: 0.75 mm)	225	Low
Xiao et al. [47]	2025	Asia	CTA (64-slices and 256-slices)	105,052	Low

### Suboccipital segment (V3) morphological variants

**Fig. 2:**
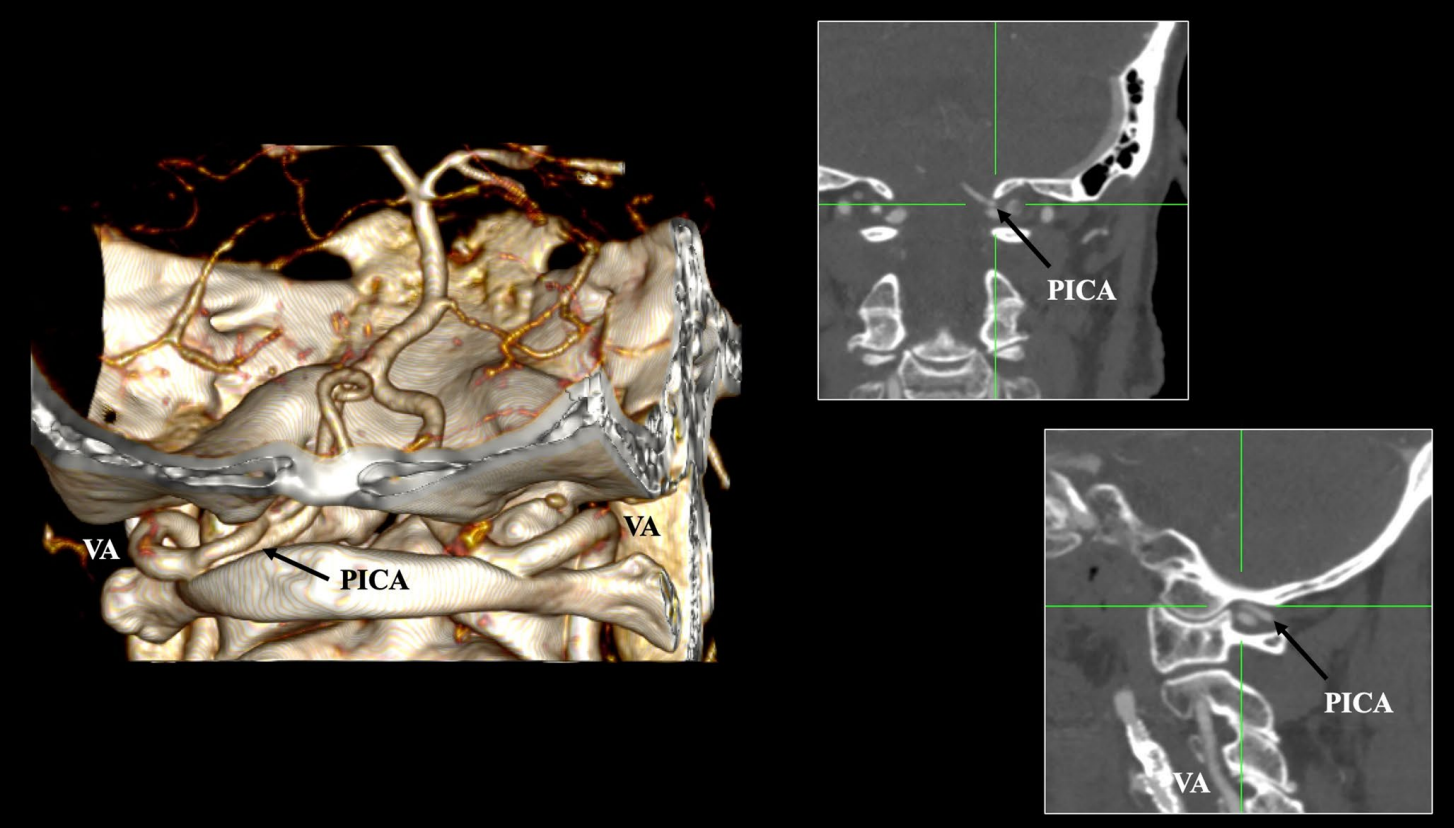
The extradural origin of the posterior inferior cerebellar artery (PICA) is depicted in three-dimensional reconstruction, sagittal, and coronal slices

The aberrant origin of the PICA was identified with a pooled prevalence of 2.69% (95% CI: 1.13–4.82) (Fig. 2). The left PICA had an aberrant origin at 0.82% and the right one at 0.69%. Bilateral occurrences were noted in less than 0.01% of cases (*p* < 0.0001). Geographical distribution (*p* = 0.2840) and imaging technique (*p* = 0.1015) did not significantly impact the pooled prevalence estimate. The DOI plot for this variant exhibited an LFK index of + 0.3, indicating no asymmetry in the pooled prevalence estimate.

**Fig. 3:**
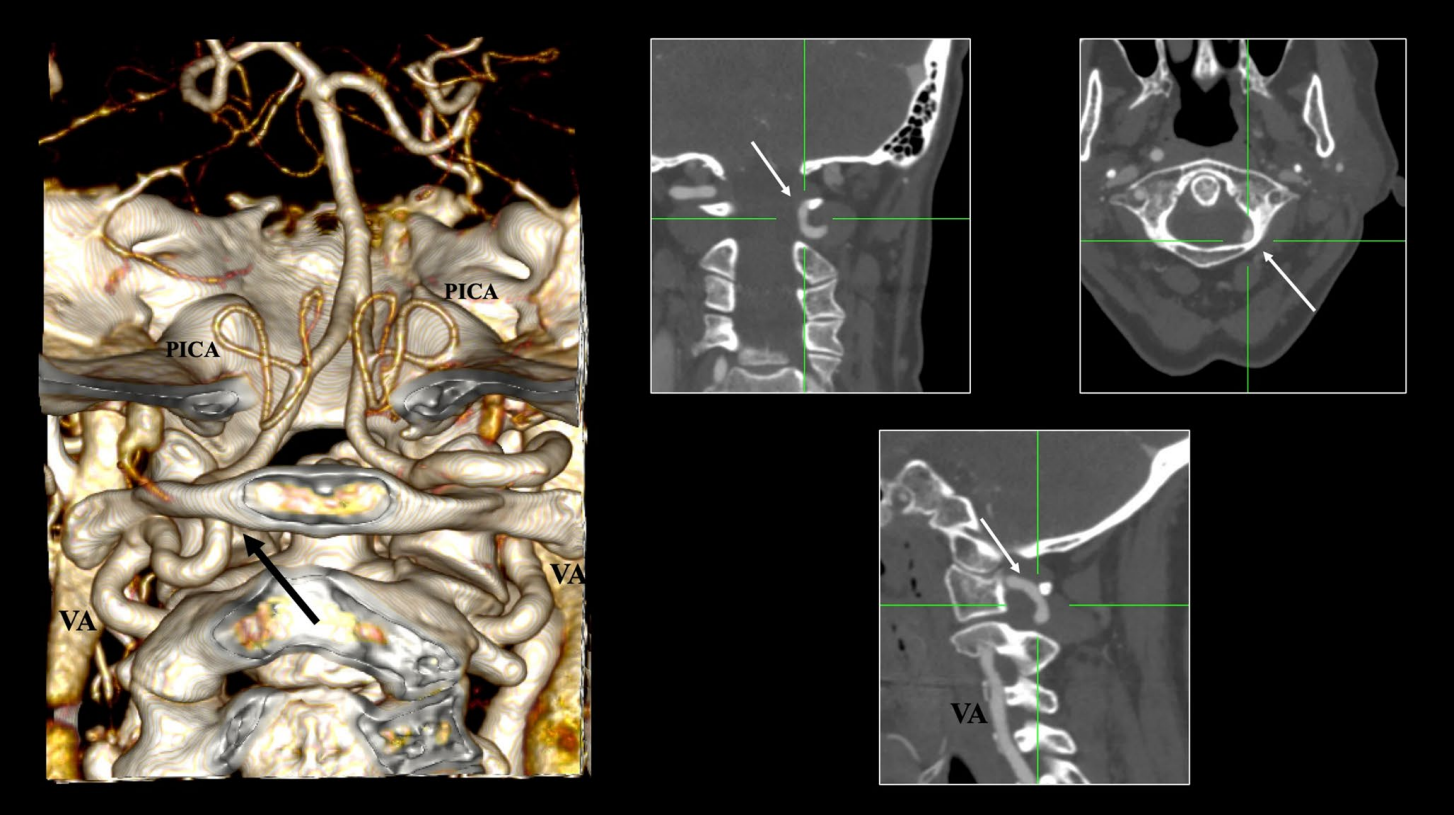
The persistent first intersegmental artery (PFIA) is indicated with black and white arrows depicted in three-dimensional reconstruction, axial, sagittal, and coronal slices. VA- vertebral artery, PICA- posterior inferior cerebellar artery

The prevalence of PFIA was estimated at a pooled prevalence of 1.17% (95% CI: 0.36–2.34) (Fig. 3). The occurrence of the right PFIA was 0.69%, and the left PFIA was 0.78%, with a bilateral occurrence observed at 0.06% (*p* = 0.1501). Nationality was identified as a significant moderator, with the Asian population presenting the highest prevalence (1.98%) and the African population exhibiting the lowest prevalence (less than 0.01%) (*p* = 0.0082). The pooled prevalence of PFIA demonstrated significant differences across imaging techniques. MRA studies revealed the highest prevalence (3.13%), whereas CTA studies showed the lowest (1.09%) (*p* = 0.009). The DOI plot for PFIA indicated an LFK index of + 3.46, demonstrating significant asymmetry in the pooled prevalence estimate.

### Intradural segment (V4) morphological variants

**Fig. 4:**
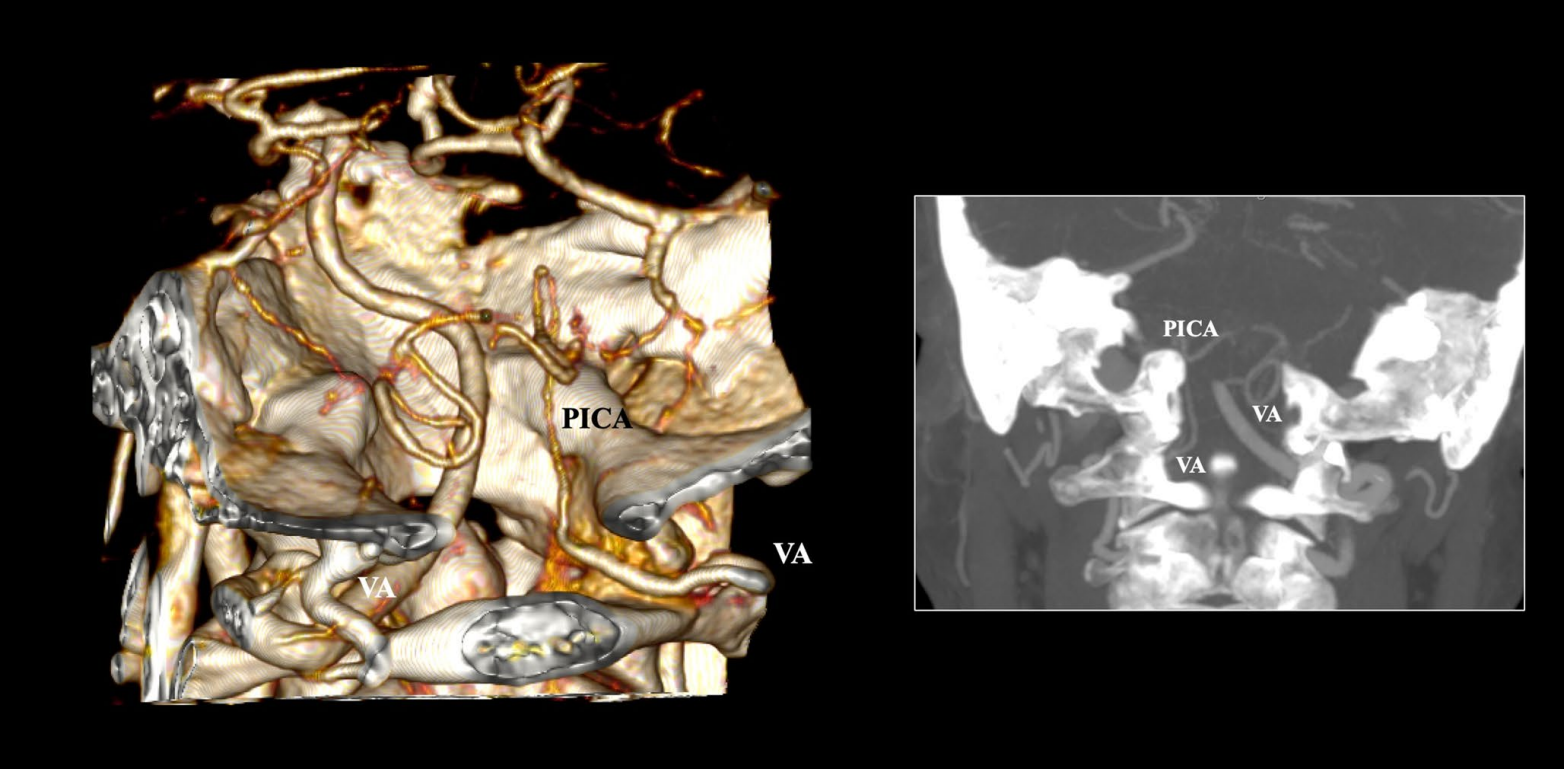
Concomitant hypoplasia and atresia of the vertebral artery (VA) on the right side with contralateral (left-sided) dominance. PICA- posterior inferior cerebellar artery. Note that the VA aplasia could be congenital aplasia or acquired occlusion

A unilateral dominance of VA was determined with a pooled prevalence of 27.45% (95% CI: 3.47–62.65). The pooled prevalence of VA hypoplasia was calculated at 13.41% (95% CI: 5.56–23.91), and that of the VA atresia was recorded at 5.39% (95% CI: 3.25–8.01) (Fig. 4).

**Fig. 5:**
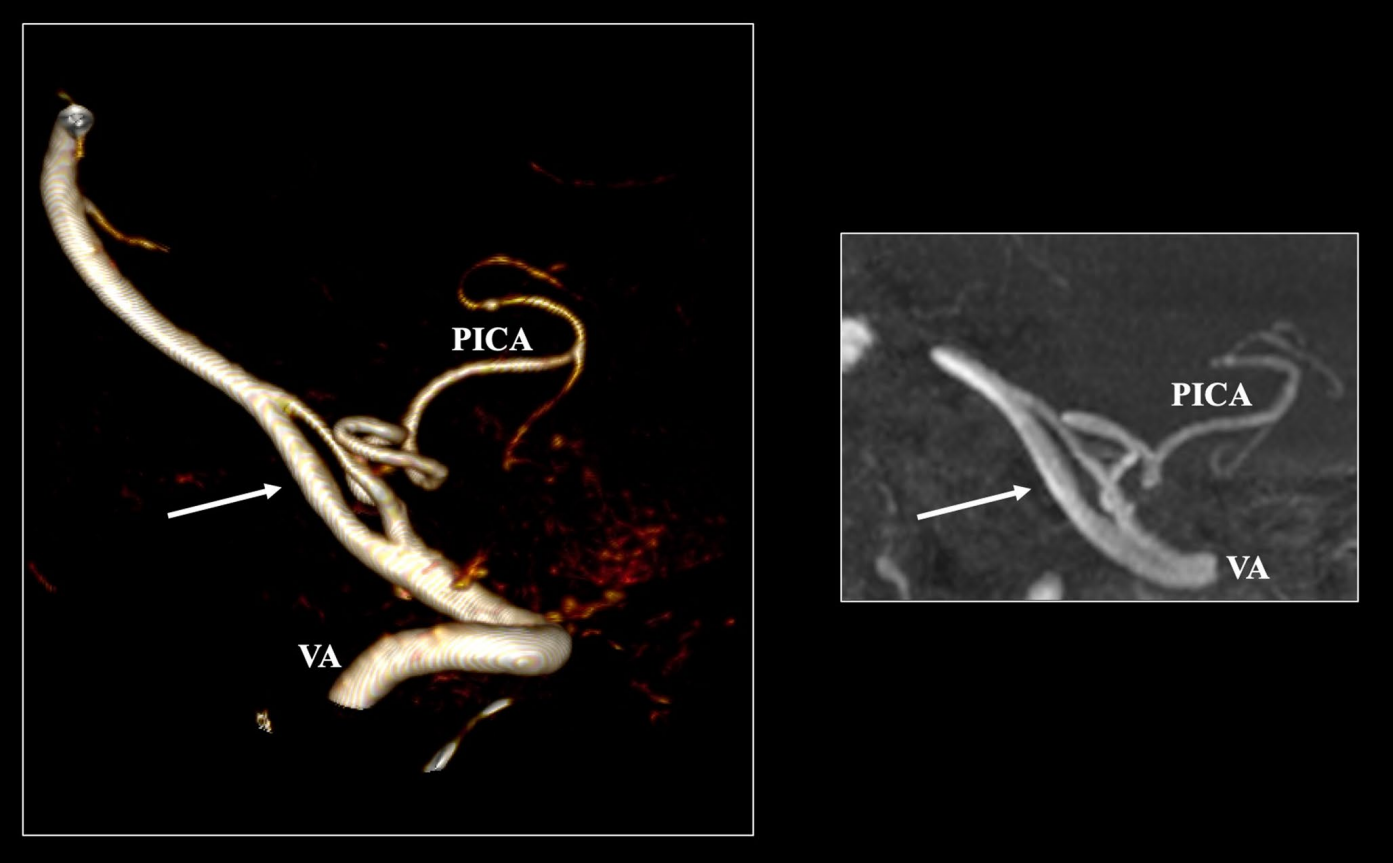
Large fenestration of the vertebral artery (VA) diagnosed through magnetic resonance angiography. The posterior inferior cerebellar artery (PICA) was arising from the fenestrated segment

The VA fenestration was calculated with a pooled prevalence of 0.30% (95% CI: 0.14–0.51) (Fig. 5). The prevalence of the RVA fenestration was 0.60%, and the LVA fenestration was 0.25%. Bilateral fenestration was recorded in less than 0.01% (*p* < 0.0001). Neither geographical distribution (*p* = 0.4912) nor imaging technique (*p* = 0.4441) affected the pooled prevalence estimate. The DOI plot illustrated an LFK index of + 0.1, indicating no asymmetry in the VA fenestration pooled prevalence estimate.

## Discussion

In the present evidence-based systematic review with meta-analysis, we documented the VA morphological variants on the suboccipital (V3) and intradural (V4) segments after calculating their pooled prevalence by considering laterality, imaging technique, and geographical area impact. In conjunction with the preceding meta-analysis concerning the V1 and V2 segments [13], the present study encompasses a comprehensive meta-analytic examination of the V3 and V4 morphological variants, presenting them in order of decreased prevalence, thereby completing the collection of VA variants. Each variant’s morphological variability and clinical significance will be further elaborated upon.

### Suboccipital segment (V3) morphological variants

The PICA typically originates from the V4 (intradural) segment. However, an aberrant origin from the V3 (suboccipital) segment has been documented, with a calculated pooled prevalence of 2.69%. In such instances, the PICA arises from an extradural location, penetrating the dura and the VA to supply blood to a portion of the cerebellum. This extradural origin can have two anatomical possibilities: at the levels of the C1 or C2 vertebrae or at the foramen magnum level [46, 48]; nonetheless, it has not been sufficiently documented from the current studies, and it was not distinguished in the current meta-analysis. Uchino and Kakehi [49] described an atypical case of PICA originating from the C2 TF, which subsequently entered the spinal canal via the C1-C2 intervertebral space. In the context of posterior approaches to the upper cervical spine or lower brainstem, it is essential to document preoperatively the presence of an aberrant PICA, as it is vulnerable to injury [46]. Additionally, PICA aneurysms may develop, and their rupture can result in subarachnoid hemorrhage; however, in the case of an aberrant PICA with aneurysm rupture, the hemorrhage would not occur within the subarachnoid space [46].

The presence of the PFIA was identified with a pooled prevalence of 1.17%, indicating that it is an infrequent variant. Our findings revealed that MRA studies have reported a higher prevalence of PFIA than CTA studies. Several remnants of the embryonic circulation exist, including the PFIA, which correlates with the VA course in the spinal canal beneath the C1 vertebra. Notable examples include persistent trigeminal (PTA) and hypoglossal arteries [8]. According to research conducted by Uchino et al. [8] and Tyagi et al. [50], the PTA variant is the most prevalent among those examined. Conversely, the meta-analysis by Brzegowy et al. [51] estimated a pooled prevalence of the PTA variant at 0.4%. The current systematic review determined that the PFIA variant is the most prevalent vessel, with a pooled prevalence of 1.17%. Therefore, this particular type of embryonic persistent vessel should be considered the most common. The PFIA poses a potential risk to posterior craniocervical junction approaches because the variant enters the spinal canal [8]. A comprehensive study conducted by Xiao et al. [47], which encompassed a total sample size of 105,052 participants, documented the clinical manifestations associated with PFIA, including the formation of aneurysms, arteriovenous malformations, and instances of cerebral infarction and hemorrhage.

### Intradural segment (V4) morphological variants

Variations, such as VA dominance, hypoplasia, and atresia, have been documented, with pooled prevalences of 27.45%, 13.41%, and 5.39%, respectively. VA dominance should be recognized when the diameter of the VA demonstrates a difference exceeding 2 mm [16]. Isaji et al. [16] noted that aberrant PICA arises from a non-dominant VA with a significantly greater occurrence (22.5%) in contrast to the dominant VA (6.25%, *p* < 0.01). VA dominance can be assessed through MRA, CTA, digital subtraction angiography (DSA), and color Doppler ultrasound [52]. However, this variant has been associated with ischemic events in the posterior circulation [52] and resultant curvature of the BA [53]. Nevertheless, it was found that an increased angle of VA dominance, also increases the BA curvature and leads to atherosclerotic plaque formation [54]. Moreover, cerebrovascular incidents within the posterior circulation have been correlated with VA atresia [55]. It is important to highlight that the imaging appearance of VA atresia could be attributed either to congenital aplasia or acquired occlusion. Liu et al. [55] discovered that patients with clinically evident cerebrovascular events exhibited a significantly higher prevalence of VA atresia than healthy individuals. Similarly, Zhu et al. [45] established an association between vertebrobasilar stroke and an increased prevalence of VA atresia.

The VA fenestration was assessed with a pooled prevalence of 0.30%, which signifies its classification as a rare variant. This particular fenestration pertains to the intradural VA segment as observed across various studies, given that descriptions of fenestration within the suboccipital segment remain absent. Intracranial fenestrations are frequently documented, with the anterior communicating artery recognized as the most prevalent site, with a pooled prevalence of 5% [3]. Lasjaunias et al. [56] delineated the presence of both true and false fenestrations, as well as duplications and arterial slits, which ought to be distinguished from one another. In instances where fenestrations are significantly large, they are categorized as duplications rather than arterial slits [24]. Radoi et al. [57] reported a case involving substantial intradural VA fenestration concomitantly with BA fenestration. Due to the extensive imaging techniques utilized in evaluating the cerebral arterial circle, these rare variants are elucidated via CTA and MRA. However, Rusu and Pop [58] documented an intradural fenestration of the VA, with the PICA arising from the proximal segment of the fenestration during dissection. From a clinical perspective, intracranial fenestrations have been associated with an elevated risk of aneurysm formation [59], arteriovenous malformations, and other developmental anomalies [60]. The fenestrated segments exhibit defects in the layers of the arterial wall at both the proximal and distal ends; consequently, these defects, along with abnormal hemodynamic stress due to altered blood flow, predispose individuals to aneurysm formation [31]. The altered hemodynamic characteristics of the wall of the fenestrated segment increase the risk of cerebrovascular events, such as ischemic stroke [61]. This fact was previously proved by imaging studies examining intracranial fenestrations’ geometrical characteristics [61, 62].

### Rarer vertebral arteries (VA) variations

Additional rare variants previously detailed in case reports can be identified concerning the V3 and V4 segments. Rusu [63] incidentally noted an ascending vermian artery originating from the V4 segment during dissection. Tudose et al. [64] documented an occipital artery arising from the V3 segment during a digital DSA of a 38-year-old female patient. Both cases emphasize the rarity of anomalous branches originating from the distal VA segments [63, 64].

### Topographical variants of the vertebral arteries (VA)

Only a limited number of variants have been documented regarding the topography of the distal VA. The most frequently cited variation is the high-riding VA, characterized as an anomalous course resulting from a C2 isthmus height of less than 5 mm and/or an internal height of less than 2 mm [65]. This variant was not included in the present meta-analysis due to its prior comprehensive documentation. Klepinowski et al. [65] estimated a pooled prevalence of 20.9% in their meta-analysis. They emphasized documenting this variant before approaching the craniocervical junction to mitigate the risk of injuries arising from its abnormal course [65]. An infrequent instance of an anomalous course of the VA was reported by Uchino and Suzuki [66]. They noted that the suboccipital segment exhibited a posterosuperior course that penetrated the jugular foramen inferior wall to enter the skull instead of traversing through the FM [66]. Recently, Rusu et al. [67] identified a similar case where the VA entered the skull base from a transoccipital parasigmoid canal. Furthermore, Davidoiu et al. [68] investigated the VA transverse and oblique course (V3-V4 segments) at the medullospinal junction. They documented a prevalence of 19.75%, indicating that such configurations are not infrequent and may present with neurological signs of medullary compression [68]. Similarly, Triantafyllou et al. [69] recorded the VA aberrant course taking a ventral path to the medullospinal junction, while they observed that this variation is correlated with unilateral VA dominance.

### Limitations

It is essential to recognize several limitations inherent in the present study. Numerous investigations have revealed a significant risk of bias, and several pooled prevalence estimates display considerable heterogeneity and wide range of confidence intervals. The LFK index of the PFIA pooled prevalence exhibits notable asymmetry, indicating a potential small study effect. These findings are consistently noted across anatomical systematic reviews and meta-analyses [14]. Sides have not reported the variants of VA dominance, hypoplasia, and atresia; laterality in the existing literature has not been estimated to estimate their respective pooled prevalences. Furthermore, it was not feasible to establish a correlation between the V3 and V4 variants with the V1 and V2 variations, as these were not reported simultaneously in the current literature. Additionally, a comparative analysis of the variants between sexes was impracticable due to insufficient eligible studies reporting such data.

## Conclusions

This evidence-based systematic review, supplemented by a meta-analysis, documented the variants of the suboccipital (V3) and intradural (V4) segments of the VA. The predominant variation observed was VA dominance, which exhibited a pooled prevalence of 27.45%, whereas VA fenestration was identified as the rarest variant, with a pooled prevalence of 0.30%. CTA, MRA, and DSA techniques can accurately characterize the distal VA morphology. Several of these morphological variants may possess clinical significance owing to their association with an increased frequency of cerebrovascular events, such as VA dominance and atresia, as well as the formation of aneurysms, particularly in cases of VA fenestration. Furthermore, a comprehensive preoperative evaluation of the VA anatomy before interventions at the craniocervical junction is essential for identifying variants that may elevate the risk of vascular injury, specifically the presence of the PFIA and the aberrant PICA.

## Data Availability

No datasets were generated or analysed during the current study.
